# The Fundus Structural and Functional Predictions of DME Patients After Anti-VEGF Treatments

**DOI:** 10.3389/fendo.2022.865211

**Published:** 2022-03-29

**Authors:** Hang Xie, Shihao Huang, Qingliang Liu, Yifan Xiang, Fabao Xu, Xiaoyan Li, Chun-Hung Chiu

**Affiliations:** ^1^ Business School, Sun Yat-Sen University, Guangzhou, China; ^2^ School of Computer Science and Engineering, Sun Yat-Sen University, Guangzhou, China; ^3^ State Key Laboratory of Ophthalmology, Zhongshan Ophthalmic Center, Sun Yat-Sen University, Guangzhou, China; ^4^ Department of Ophthalmology, Qilu Hospital, Cheeloo College of Medicine, Shandong University, Jinan, China

**Keywords:** diabetic macular edema, optical coherence tomography, visual acuity, clinical effectiveness, prognosis prediction

## Abstract

Diabetic retinopathy (DR) is an important complication with a high incidence of 34.6% in the diabetic populations. DR could finally lead to vision impairment without effective interventions, during which, diabetic macular edema (DME) is a key phase causing visual loss. Up to date, antivascular endothelial growth factor (anti-VEGF) therapy is the first-line treatment for DME which has achieved relatively better clinical outcomes than traditional treatments. However, there are several kinds of anti-VEGF medicines, and patients are sensitive to different anti-VEGF treatments. In addition, its effectiveness is unstable. Considering the patients’ need to accept continual anti-VEGF treatments and its price is comparatively high, it is clinically important to predict the prognosis after different anti-VEGF treatments. In our research, we used the demographic and clinical data of 254 DME patients and 2,763 optical coherence tomography (OCT) images from three countries to predict the fundus structural and functional parameters and treatment plan in 6 months after different anti-VEGF treatments. Eight baseline features combined with 11 models were applied to conduct seven prediction tasks. Accuracy (ACC), the area under curve (AUC), mean absolute error (MAE), and mean square error (MSE) were respectively used to evaluate the classification and regression tasks. The ACC and AUC of structural predictions of retinal pigment epithelial detachment were close to 1.000. The MAE and MSE of visual acuity predictions were nearly 0.3 to 0.4 logMAR. The ACC of treatment plan regarding continuous injection was approaching 70%. Our research has achieved great performance in the predictions of fundus structural and functional parameters as well as treatment plan, which can help ophthalmologists improve the treatment compliance of DME patients.

## Introduction

Diabetes is a global public health issue (1). According to published results, 1 in 11 adults had diabetes worldwide in 2015, and the diabetic population will increase to 642 million by 2040 (2–4). Diabetic retinopathy (DR) is an important complication with a high incidence of 24.7% to 37.5% in the diabetic populations (5). DR could gradually progress and finally lead to vision impairment without effective intervention, during which diabetic macular edema (DME) is a key phase causing visual loss. The morbidity of DME is 3.1% to 7.9% in the diabetic populations, and most of them need prompt and effective treatment to avoid severe visual impairment (6).

Up to date, antivascular endothelial growth factor (anti-VEGF) therapy is the first-line treatment for DME and has achieved relatively better clinical outcomes than traditional treatments of retinal photocoagulation and surgery (7–9). There are several kinds of anti-VEGF medicines, involving bevacizumab, ranibizumab, aflibercept, and so on. However, patients are sensitive to different anti-VEGF treatments and its effectiveness is unstable (10–12). Considering that patients need to accept repetitive anti-VEGF treatments and its price is comparatively high, it is clinically important to predict the prognosis after different anti-VEGF treatments (13, 14).

In our study, we established intelligent models for fundus structural and functional predictions of DME patients after different anti-VEGF treatments. Based on the multinational data, we applied several algorithms to predict the functional parameter of visual acuity (VA), structural parameters of central retinal thickness (CRT) and other four parameters, and clinical advice of continuing injection (CI) 6 months in advance. Our models have achieved great performance in different prediction tasks, which can help ophthalmologists make treatment plans for DME patients and provide research basis for other retinopathies.

## Materials and Methods

The data were downloaded from the open dataset provided by the Asia Pacific Tele-Ophthalmology Society (APTOS) and the Department of Ophthalmology, Qilu Hospital, Shandong University, from October 2018 to May 2021. The APTOS data were applied to train and test the models. The Qilu data were applied for external validation. Our ethics committee ruled that written informed consent was not required because our study was retrospective in nature and all the images were fully anonymized. Moreover, this study adhered to the tenets of the Declaration of Helsinki (2020KYPJ024).

## Data Collection

The APTOS data were obtained from the Rajavithi Hospital of Thailand and the Aravind Eye Hospital of India, then labeled by the Zhongshan Ophthalmic Center of China. The Qilu data were extracted from the clinical records. Only patients diagnosed with DME and accepting anti-VEGF treatments are enrolled. The inclusion criteria were as follows (1): aged greater or equal to 18 years; (2) diagnosed with DME; and (3) accepting consecutive monthly anti-VEGF therapy of bevacizumab, aflibercept, conbercept, ranibizumab, or combination for 6 months. The exclusion criteria were as follows: (1) presence of any other retinal diseases including age-related macular degeneration (AMD), retinal vein occlusion (RVO), polypoidal choroidal vasculopathy (PCV), and so on; (2) low image quality caused by media opacities; or (3) an abnormal signal strength index of images. The follow-up points were at 6 months after anti-VEGF therapy.

The demographic information of age and gender were recorded. Pretherapeutic visual acuity (pre-VA) and visual acuity (VA) after anti-VEGF therapy for 6 months were tested. Based on the optical coherence tomography (OCT) of patients before and after anti-VEGF therapy, five imaging characteristics of central retinal thickness (CRT), and presence of retinal interlayer fluid (RIF), subretinal pigment epithelium fluid (SRF), retinal pigment epithelial detachment (PED), and retinal hyperreflexia (RHF) were measured and recorded by ophthalmologists with clinical work experience of more than 5 years. The clinical decision label of suggesting continuous injection (CI) of anti-VEGF medicine was made based on its therapeutic effect after 6 months according to changes of VA and imaging characteristics, which was labeled by professors with clinical work experience of more than 10 years. As the appearance and treatment of binocular DME are usually asynchronous, we treated each eye as a separate case in data collection.

## Model Training and Evaluation

**Figure 1:**
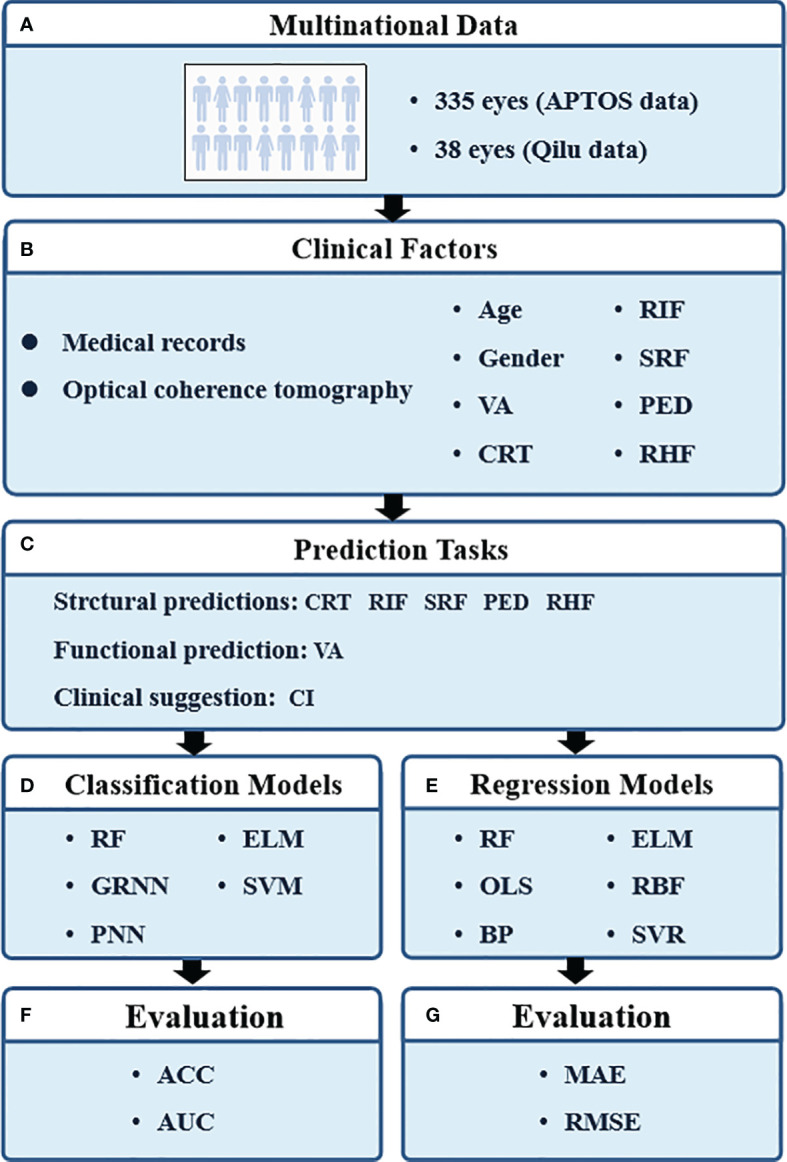
The pipeline of our study. VA, visual acuity; CRT, central retinal thickness; RIF, retinal interlayer fluid; SRF, subretinal pigment epithelium fluid; PED, retinal pigment epithelial detachment; RHF, retinal hyperreflexia; CI, continuous injection; RF, random forest; GRNN, generalized regression neural network; PNN, probabilistic neural network; ELM, extreme learning machine; SVM, support vector machines; OLS, ordinary least squares; BP, back propagation network; RBF, radial basis function network; SVR, support vector regression; ACC, classification accuracy; AUC, area under curve; MAE, mean absolute error; RMSE, root mean square error.

The research procedure is shown in **Figure 1**. Eight parameters were applied to train the prediction models, including age, gender, pre-VA, pre-CRT, pre-RIF, pre-SRF, pre-PED, and pre-RHF at baseline. Five classification prediction models were constructed to predict the CI and the presence of RIF, SRF, PED, and RHF. Six regression prediction models were established to predict VA and CRT 6 months in advance.

The classification prediction models included classical random forest (RF), generalized regression neural network (GRNN), probabilistic neural network (PNN), extreme learning machine (ELM), and support vector machine (SVM) modeling. Also, their classification performances were evaluated by classification accuracy (ACC) and area under the curve (AUC). ACC is defined as the proportion of the sum of true-positive samples (TP) and the true-negative samples (TN) to the total number of samples (*N*) to be predicted, as follows,


$$\text{ACC}\;=\;\frac{TP+TN}{N}.$$

AUC represents the area under the ROC curve, and AUC takes values between 0 and 1, where a larger AUC indicates a better model performance.

The regression prediction models contained RF, ordinary least squares (OLS), back propagation (BP) network, ELM, radial basis function (RBF) network, and support vector regression (SVR) modeling. The prediction performances of regression models were quantified with the mean absolute error (MAE) and root mean square error (RMSE). The MAE is defined as the average value of the absolute error of the prediction results, which directly reflects the deviation of the predicted values from the actual values, as follows,


$$\text{MAE}\;=\;\frac{1}{N}\sum_{i=1}^{N}|{\tilde{y}}_{i}-y_{i}|,$$

where $\tilde{y_{i}}$ and *y_i_* represent the model prediction value and the real value for the *i*th sample, respectively. The RMSE is the square root of mean square error (MSE), which is calculated as the average value of the square of the error of the prediction results, as follows,


$$\text{RMSE}\;=\;\sqrt{\frac{1}{N}\sum_{i=1}^{N}{\left({\tilde{y}}_{i}-y_{i}\right)}^{2}}.$$

## Results

Of 216 patients, 335 eyes were included from APTOS data and 38 eyes of 38 patients were enrolled form Qilu data (**Table 1**). The DME patients were aged 26 to 84 years old with a mean age of nearly 56 years old. One hundred fourteen (34.0%) patients and 22 (57.9%) patients were men in the APTOS and Qilu data, respectively. The pre-VAs were 0.74 ± 0.60 (0–3) logMAR and 1.20 ± 0.63 (0–3) logMAR in two datasets (*p* < 0.001). The pre-CRTs ranged from 151 to 1,345 μm with mean values of 426.71 ± 177.88, 415.21 ± 131.98, and 699.87 ± 921.63 μm in the training, test, and external validation groups, respectively. The statistical differences of all characteristics before and after treatments between the APTOS and Qilu data are shown in **Table 1**. Except for the baseline age, pre-VA and pre-RHF, there was no statistical difference between two datasets. DME patients of three groups showed VA improvement, CRT decrease, and percentage changes of RIF, SRF, PED, and RHF, which are described in detail in **Table 1**.

**Table 1:** The characteristics of patients according to groups.

Characteristics	Training group	Test group	External validation	*p*-values
Patients	182	34	38	
Gender (men)	98 (53.85%)	16 (47.06%)	22 (57.89%)	0.726
Age (year)	56.55 ± 9.98	57.59 ± 10.27	61.84 ± 11.19	0.005
Eyes	301	34	38	
Baseline				
Pre-VA (logMAR)	0.74 ± 0.60	0.60 ± 0.42	1.20 ± 0.63	<0.001
Pre-CRT (μm)	426.71 ± 177.88	415.21 ± 131.98	699.87 ± 921.63	0.08
Pre-RIF	243 (80.73%)	27 (79.41%)	34 (89.47%)	0.355
Pre-SRF	98 (32.56%)	10 (29.41%)	18 (47.37%)	0.260
Pre-PED	26 (8.64%)	1 (2.94%)	2 (5.26%)	0.609
Pre-RHF	205 (68.11%)	20 (58.82%)	20 (52.63%)	0.039
Posttreatment				
VA (logMAR)	0.69 ± 0.59	0.52 ± 0.31	0.87 ± 0.64	0.184
CRT (μm)	391.08 ± 157.26	392.56 ± 140.09	355.55 ± 132.69	0.243
RIF	242 (80.40%)	25 (73.53%)	29 (76.32%)	0.359
SRF	66 (21.93%)	4 (11.76%)	8 (21.05%)	0.676
PED	26 (8.64%)	1 (2.94%)	2 (5.26%)	0.638
RHF	202 (67.11%)	19 (55.88%)	20 (52.63%)	0.056
CI	203 (67.44%)	25 (73.53%)	21 (55.26%)	0.052

p-values showed the statistical differences of characteristics between the APTOS and Qilu data.

VA, visual acuity; CRT, central retinal thickness; RIF, retinal interlayer fluid; SRF, subretinal pigment epithelium fluid; PED, retinal pigment epithelial detachment; RHF, retinal hyperreflexia; CI, continuous injection.

Five structural prediction models obtained ACCs and AUCs at different levels, of which the RF and SVM models achieved best performances (**Table 2**). In the internal test, ACCs of presence predictions regarding RIF, SRF, PED, and RHF were 0.902, 0.825, 0.937, and 0.939 with the RF model and 0.912, 0.787, 0.965, and 0.948 with the SVM model. AUCs of presence predictions regarding RIF, SRF, PED, and RHF were 0.844, 0.645, 0.772, and 0.891 with the RF model and 0.865, 0.525, 0.906, and 0.890 with the SVM model. In the external validation, these two models also obtained high-level accuracies that ACCs of presence predictions regarding RIF, SRF, PED, and RHF were 0.858, 0.783, 1.000, and 0.917 with the RF model and 0.868, 0.777, 1.000, and 0.895 with the SVM model. AUCs of presence predictions regarding RIF, SRF, PED, and RHF were 0.782, 0.654, 1.000, and 0.944 with the RF model and 0.812, 0.622, 1.000, and 0.911 with the SVM model, which have achieved excellent performance in the PED predictions with an AUC of 1.000 in external validation. The predictions of RIF and RHF obtained high accuracies of more than 0.900 as well. In the clinical suggestions of CI predictions, the accuracy is close to 0.700. In the internal test, the ACCs of CI predictions were 0.671 with the RF model and 0.697 with the SVM model. The AUCs were 0.607 with the RF model and 0.634 with the SVM model. In the external validation, ACCs were 0.674 and 0.656 and AUCs were 0.649 and 0.671 respectively in the RF and SVM models.

**Table 2:** The prediction performances of the best two models in classification and regression tasks.

Classification models	RF				SVM			
	ACC		AUC		ACC		AUC	
Factors	Test	Validation	Test	Validation	Test	Validation	Test	Validation
RIF	0.901	0.857	0.843	0.781	0.911	0.868	0.864	0.812
SRF	0.824	0.782	0.645	0.654	0.787	0.776	0.525	0.622
PED	0.936	1.000	0.772	1.000	0.964	1.000	0.905	1.000
RHF	0.938	0.916	0.890	0.943	0.947	0.894	0.887	0.911
CI	0.671	0.607	0.674	0.656	0.697	0.634	0.649	0.671
**Regression models**	**SVR**				**RF**			
	**MAE**		**RMSE**		**MAE**		**RMSE**	
**Factors**	**Test**	**Validation**	**Test**	**Validation**	**Test**	**Validation**	**Test**	**Validation**
VA (logMAR)	0.262	0.387	0.414	0.511	0.302	0.485	0.446	0.585
CRT (μm)	99.91	162.34	140.36	330.86	94.86	131.26	128.94	159.24

RF, random forest; SVM, support vector machines; SVR, support vector regression; ACC, classification accuracy; AUC, area under curve; MAE, mean absolute error; RMSE, root mean square error; RIF, retinal interlayer fluid; SRF, subretinal pigment epithelium fluid; PED, retinal pigment epithelial detachment; RHF, retinal hyperreflexia; CI, continuous injection; VA, visual acuity; CRT, central retinal thickness.

The SVR and RF models obtained the best performances in VA and CRT predictions (**Table 2**). MAEs of VA predictions were 0.262 and 0.302 logMAR in internal tests and 0.387 and 0.485 logMAR in external validations. Prediction errors were equivalent to nearly three lines of visual chart. Predictions errors of CRT were exhibited in **Table 2**. Prediction errors of MAE and RMSE were close to 100 to 150 μm.

**Figure 2:**
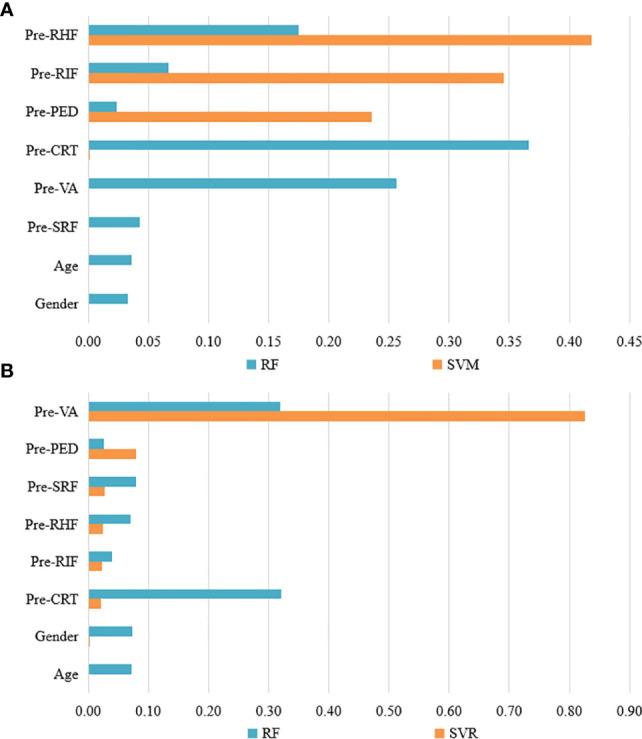
The feature weights of CI and VA predictions of the best two models. **(A)** The feature weights of CI predictions with RF and SVW models. **(B)** The feature weights of VA predictions with RF and SVR models. VA, visual acuity; CI, continuous injection; CRT, central retinal thickness; RIF, retinal interlayer fluid; SRF, sub retinal pigment epithelium fluid; PED, retinal pigment epithelial detachment; RHF, retinal hyperreflexia; RF, random forest; SVM, support vector machines; SVR, support vector regression.

Feature weights of CI and VA predictions of the best two models are indicated in **Figure 2**. Two models showed different weight patterns in the same tasks. In the CI predictions, the most important features were pre-RIF (0.345), pre-PED (0.236), and pre-RHF (0.419) according to the SVM model. However, in the RF model, the most important features were pre-VA (0.257), pre-CRT (0.366), and pre-RHF (0.175). In the VA predictions, the pre-VA had the highest weights of 0.826 and 0.319 in the SVR and RF models, respectively. The pre-CRT (0.321) also played an important role in VA predictions based on the RF model.

## Discussion

Our study established classification and prediction models with multinational data to predict the treatment effects of anti-VEGF therapy for DME patients. The models have achieved excellent performance in prediction tasks of structural and functional parameters as well as clinical suggestions 6 months in advance, which can help ophthalmologists with DME patients.

DME is an important stage of RD, which needs timely treatment, otherwise may cause severe visual impairment (15). Anti-VEGF treatment is the first-line treatment of DME (16, 17). Increasing researches were conducted to explore the clinical effect and limitation of anti-VEGF treatment (11, 18–21). Although anti-VEGF treatment was proved to function better than previous therapies of laser therapy and steroid therapy, it had some limitations as well (22–24). Patients need long-term and consecutive anti-VEGF injections, and the complication morbidity will increase with the treatment progress (7, 25, 26). The potential ocular complications include entophthalmia, glaucoma, and retinal detachment, which can lead to a more severe visual loss (27, 28). Consequently, to predict the therapeutic effect of anti-VEGF treatment can provide basis to make intervention plan for both ophthalmologists and patients. The functional prediction of VA and treatment plan prediction of CI both serve as key roles in treatment guidance. The prediction of CI is an impossible task for human ophthalmologists, even for experienced fundus professors. Our models achieved nearly 70% accuracy in the CI predictions, which provided a new sight in the understanding treatment effectiveness.

In our research, we finished the structural prediction tasks of five parameters, including CRT, RIF, SRF, PED, and RHF, which are all relatively important in the DME process and prognosis (29). Thick CRT and the presence of RIF, SRF, PED, and RHF all indicate there are still effusion and exudation in the lesion areas and that the prognosis is unsatisfactory (12, 30, 31). Previous studies have published relative prediction results, and some studies accurately predicted the VA and CRT of DME patients after anti-VEGF treatment by clinical information and OCT data (32–35). Honghua Yu and his team have published results predicting the VA and CRT of DME patients 1 month after anti-VEGF treatments (36). Our research has achieved earlier predictions of 6 months with more parameters. Our models obtained high accuracies in the presence predictions of more structural parameters, including RIF, SRF, PED, and RHF, especially the presence of PED, which provided more information about the treatment effects. Moreover, some other retinopathies, such as central serous chorioretinopathy and age-related macular degeneration, have similar pathological changes (37–39). Our results may offer a research basis for the intelligent structural predictions of other retinopathies.

The two best models in VA and CI predictions exhibited different weight patterns according to the analysis. Based on the SVM models, the patients showing the presence of pre-RIF, pre-PED, and pre-RHF may need anti-VEGF treatment more and may obtain higher clinical effectiveness with CI. The presence of these structural lesions indicates there are chronic vascular leakage and fluid accumulation, which may cause angiogenesis (40, 41). As a result, anti-VEGF treatment is beneficial to patients with these structural lesions. Our study found the clinical basis to support the continuous injection of anti-VEGF medicine, which may provide a new idea for studied on the effectiveness of anti-VEGF treatment.

## Limitation

Some limitations of our research should be considered. Larger samples of DME patients are needed to enrich the data and to improve the prediction accuracy. Moreover, data form more external validations are necessary to test the stability of prediction models. Additionally, the intelligent models for predicting the effect of different anti-VEGF treatments are essential to assist doctors in making a clinical plan.

## Data Availability Statement

The raw data supporting the conclusions of this article will be made available by the authors, without undue reservation.

## Ethics Statement

Our ethics committee ruled that written informed consent was not required because our study was retrospective in nature and all the images were fully anonymized. Moreover, this study adhered to the tenets of the Declaration of Helsinki (2020KYPJ024). The patients/participants provided their written informed consent to participate in this study.

## Author Contributions

HX, SH, and C-HC conceived and designed the experiments. FX and XL collected the data. YX, FX, and XL labeled the data. SH and QL performed the experiments and analyzed the data. HX wrote the paper, and SH and CHC revised it. All authors read and approved the final manuscript.

## Funding

This study was funded by the National Natural Science Foundation of China (Grant No. 71971224 and 71721001) and Guangdong Basic and Applied Basic Research Foundation (Grant No. 2020A1515011081). The sponsors of the study played no role in the study protocol design; data collection, analysis, or interpretation; manuscript preparation; or the decision to submit the manuscript for publication.

## Conflict of Interest

The authors declare that the research was conducted in the absence of any commercial or financial relationships that could be construed as a potential conflict of interest.

## Publisher’s Note

All claims expressed in this article are solely those of the authors and do not necessarily represent those of their affiliated organizations, or those of the publisher, the editors and the reviewers. Any product that may be evaluated in this article, or claim that may be made by its manufacturer, is not guaranteed or endorsed by the publisher.
